# African swine fever virus MGF360-4L protein attenuates type I interferon response by suppressing the phosphorylation of IRF3

**DOI:** 10.3389/fimmu.2024.1382675

**Published:** 2024-09-13

**Authors:** Zhen Wang, Yuheng He, Ying Huang, Wenzhu Zhai, Chunhao Tao, Yuanyuan Chu, Zhongbao Pang, Hongfei Zhu, Peng Zhao, Hong Jia

**Affiliations:** ^1^ College of Veterinary Medicine, Shandong Agricultural University, Tai’an, Shandong, China; ^2^ Institute of Animal Sciences, Chinese Academy of Agricultural Sciences, Beijing, China

**Keywords:** African swine fever virus, MGF360-4L, type I interferon, suppress, phosphorylation

## Abstract

African swine fever (ASF) is a highly contagious and lethal disease of swine caused by African swine fever virus (ASFV), and the mortality rate caused by virulent stains can approach 100%. Many ASFV viral proteins suppress the interferon production to evade the host’s innate immune responses. However, whether ASFV MGF360-4L could inhibit type I interferon (IFN-I) signaling pathway and the underlying molecular mechanisms remain unknown. Our study, indicated that ASFV MGF360-4L could negatively regulates the cGAS-STING mediated IFN-I signaling pathway. Overexpressing ASFV MGF360-4L could inhibit the cGAS/STING signaling pathway by inhibiting the interferon-β promoter activity, which was induced by cGAS/STING, TBK1, and IRF3-5D, and further reduced the transcriptional levels of ISG15, ISG54, ISG56, STAT1, STAT2, and TYK2. Confocal microscopy and immunoprecipitation revealed that MGF360-4L co-localized and interacted with IRF3, and WB revealed that ASFV MGF360-4L suppressed the phosphorylation of IRF3. 4L-F2 (75-162 aa) and 4L-F3 (146-387 aa) were the crucial immunosuppressive domains and sites. Altogether, our study reveals ASFV MGF360-4L inhibited cGAS‐STING mediated IFN-I signaling pathways, which provides insights into an evasion strategy of ASFV involving in host’s innate immune responses.

## 1 Introduction

African swine fever virus (ASFV) is a DNA arbovirus of swine which is the only member of the genus Asfivirus in the family Asfarviridae (1). The genome consists of a linear double-stranded DNA molecule ranging from 170 to 190 kb, encoding 150 to 200 proteins, including 68 structural proteins and more than 100 nonstructural proteins (2, 3). Numerous studies have shown that Many proteins of ASFV play important roles in viral assembly, replication, virus–host interaction, and immune evasion (4–9). African swine fever (ASF) is a an acute hemorrhagic and highly contagious disease of domestic and wild pigs caused by ASFV, with mortality of rates up to 100%. In the early 1920s, ASF was first reported in Kenya (10), then spread to most sub-Saharan African countries, the Russian Federation, Transcaucasus, some Eastern and Central European countries, Sardinia, and Southeast and East Asia (11, 12). In 2018, ASF was introduced into China, caused substantial economic losses and threatening the global pig industry (13, 14). So it has been listed as a notifiable disease by the World Organization for Animal Health (9, 15, 16), formerly known as the Office International des Epizooties. To date, no safe and effective commercial vaccines are available for ASF. Thus, the prevention and control of ASF mainly depend on strict biosecurity measures, which have economic burden. So, it is urgently and necessarily to develop a safe and effective ASF vaccine.

As the first line of defense against invading pathogens, innate immune responses rapidly detect pathogens and initiate a series of measures to kill them. Type I interferon (IFN) is produced upon recognition of pathogen-associated molecular patterns through host pattern recognition receptors (17). Cells have various strategies to combat pathogenic invasion. When DNA viruses infect the host cell, cyclic GMP-AMP synthase (cGAS) recognizes and binds to viral genomic DNA, subsequently catalyzing the synthesis of the second messenger molecule cGAMP. The dimerized stimulator of the interferon gene (STING) binds to cGAMP, causing a conformational change (18, 19). STING recruits TANK-binding kinase (TBK1) and traffics from the endoplasmic reticulum to a perinuclear endosomal compartment, leading to the activation of IFN regulatory factor 3 (IRF3) and resulting in IFN-β production (20). As ASFV is a double-stranded DNA virus, it can be sensed by cGAS (21). However, ASFV has evolved multiple strategies to inhibit the production of IFN-I by negatively regulating the antiviral cGAS-STING signaling pathway; numerous studies have demonstrated this. For example, the ASFV MGF360-14L protein was found to promote the degradation of IRF3 through TRIM21, thus negatively regulating IFN-I signaling (22). Cui et al. proved that the ASFV M1249L protein antagonizes IFN-I production by suppressing the phosphorylation of TBK1 and degrading IRF3 (23). Proteins such as E120R (24, 25), DP96R (26), and MGF505-11R (27) also inhibit IFN-β production by negatively regulating the cGAS/STING signaling pathway. Multigene family 360 (MGF360) and MGF530/505 of ASFV are involved in regulating IFN-I. The members of MGF360 are associated with virus virulence and, consequently, have been targeted for the development of live attenuated vaccine (28, 29). ASFV MGF360-4L, which contains 387 amino acids, is a member of the MGF360 family. Previous research has shown that MGF360-4L inhibits the cGAS/STING-mediated activation of the IFN-β promoter through a double luciferase assay. After adapting to the vero cells, BA71V became a non-virulent ASFV, the genomic changes include the deletion of the MGF360-4L. However, the molecular mechanism of host antiviral innate immune responses has not been clarified.

In this study, we found that overexpression of MGF360-4L could significantly inhibit the activation of IFN-β promoter activity and suppress the mRNA levels of IFN-β and interferon-stimulated genes (ISGs). Further research showed that MGF360-4L could interact with IRF3 and suppress the phosphorylation of IRF3, thereby inhibiting the cGAS‐STING mediated IFN-I signaling pathways. Our findings reveal a novel function of ASFV MGF360-4L and provide a new candidate gene for ASFV live-attenuated vaccines.

## 2 Materials and methods

### 2.1 Viruses and cells

Human embryonic kidney 293T (HEK293T) and porcine kidney 15 (PK-15) cells were obtained from Type Culture Collection of Chinese Academy of Science and cultured in Dulbecco’s modified Eagle’s medium supplemented with 10% fetal bovine serum, 100 U/ml penicillin, and 100 μg/ml streptomycin at 37 °C with 5% CO_2_. Vesicular Stomatitis Virus (VSV-GFP) was a gift from Dr Liqi Zhu of Yangzhou University, VSV-GFP is tagged with GFP and stably express green fluorescence.

### 2.2 Antibodies and reagents

Monoclonal rabbit anti-hemagglutinin, anti-TBK1/NAK, anti-phospho-TBK1/NAK (Ser172), anti-IRF-3, anti-phospho-IRF-3 (Ser396), anti-glyceraldehyde 3-phosphate dehydrogenase (GAPDH), and horseradish peroxidase-conjugated goat anti-rabbit IgG were purchased from Cell Signaling Technology (USA). Rabbit monoclonal anti-flag and anti-GFP were obtained from Proteintech (USA). Rabbit IgG and mouse IgG were supplied by Beyotime (China). Alexa Fluor 594-conjugated goat anti-rabbit IgG antibody and Alexa Fluor 488-conjugated goat anti-mouse IgG antibody were purchased from ZSGB-BIO (China). 2′3′-cGAMP was acquired from MedChemExpress (USA). poly (dA:dT) was bought from InvivoGen (Hong Kong, China). jetPRIME transfection reagent and double-luciferase reporter assay kit were obtained from Polyplus Transfection (France) and TransGen (China), respectively.

### 2.3 Plasmids

The full length sequence of ASFV MGF360-4L was synthesized, referencing the genome of ASFV CADC-HN09 (GenBank submission No. MZ614662.1), and cloned into the p3×Flag-CMV-7.1 vector by GenScript (China). The hemagglutinin (HA)-tagged cGAS, STING, and IRF3 were generated using the methods previously described by Wang et al. (26). The IFN-β and NF-κB promoter luciferase reporter plasmids, as well as the pRL-TK plasmids, were purchased from Genomeditech (China). The Flag-tagged TBK1 expression plasmid was stored in our laboratory.

### 2.4 Dual-luciferase reporter assay

HEK293T cells were seeded into 48-well plates and cultured overnight to 80% confluence. They were then co-transfected with MGF360-4L or control vector plasmids, along with IFN-β or NF-κB (20 ng/well) promoter and pRL-TK (2 ng/well) plasmids as an internal control. After 24 h of transfection, the cells were collected and lysed. The whole-cell lysates were measured using a double-luciferase reporter assay kit (TransGen Biotech, China), following the manufacturer’s protocols. The relative luciferase activity was determined by calculating the ratio of firefly luciferase to Renilla luciferase activity.

### 2.5 RNA extraction and quantitative reverse transcription polymerase chain reaction

Total RNA was extracted from HEK293T cells and PK-15 cells using the TRIzol reagent (Thermo Fisher Scientific), following the manufacturer’s instructions, and was reverse transcribed using the RT master mix (TaKaRa). The reverse transcription products were then detected using the ABI 7900HT real-time PCR system with the SYBR Green Master Mix (TOYOBO). The relative mRNA levels of the target genes were normalized to the pig GAPDH or human GAPDH mRNA levels. The abundance of individual mRNA transcripts was calculated using the 2^-ΔΔCT^ method. The primers listed in **Table 1** are used for the quantitative reverse transcription polymerase chain reaction (RT-qPCR).

**Table 1:** Primers used for qPCR in this study.

Prime	Sequence (5’-3’)	Target gene
pIFN-β-F	GTGGAACTTGATGGGCAGAT	Porcine IFN-β gene
pIFN-β-R	TTCCTCCTCCATGATTTCCTC	
pGAPDH-F	CGTCCCTGAGACACGATGGT	Porcine GAPDH gene
pGAPDH-R	GGAACATGTAGACCATGTAG	
hISG56-F	GCTTTCAAATCCCTTCCGCTAT	Human ISG56 gene
hISG56-R	GCCTTGGCCCGTTCATAAT	
hISG54-F	CACCTCTGGACTGGCAATAGC	Human ISG54 gene
hISG54-R	GTCAGGATTCAGCCGAATGG	
hISG15-F	GGGACCTGACGGTGAAGATG	Human ISG15 gene
hISG15-R	CGCCGATCTTCTGGGTGAT	
hIFN-β-F	ATGACCAACAAGTGTCTCCTCC	Human IFN-*β* gene
hIFN-*β*-R	GCTCATGGAAAGAGCTGTAGTG	
hGAPDH-F	TCATGACCACAGTCCATGCC	Human GAPDH gene
hGAPDH-R	GGATGACCTTGCCCACAGCC	
hJAK1-F	CTCTCTGTCACAACCTCTTCGC	Human JAK1 gene
hJAK1-R	TTGGTAAAGTAGAACCTCATGCG	
hSTAT1-F	AATGTGAAGGACAAGGTTATG	Human STAT1 gene
hSTAT1-R	TTGGTCTCGTGTTCTCTG	
hSTAT2-F	AATCATCCGCCATTACCA	HumanSTAT2 gene
hSTAT2-R	AGTTCATCCACCTGTCTATT	
hSTAT3-F	GGAGAAACAGGATGGCCCAA	Human STAT3 gene
hSTAT3-R	ATCCAAGGGGCCAGAAACTG	
hTYK2-F	CAGCCCCGTGTTCTGGTATG	Human TYK2 gene
hTYK2-R VSV-F VSV-R	GAAAGGACGCCTCTGTCTCC TGCAAGGAAAGCATTGAACAA GAGGAGTCACCTGGACAATCAC	VSV G gene

### 2.6 Co-immunoprecipitation assays

HEK293T cells were seeded into six-well plates and cultured overnight to 80% confluence. They were then co-transfected with MGF360-4L-Flag and IRF3-HA. After 24 h of transfection, the cells were lysed on ice using Pierce™ IP Lysis Buffer (Thermo Fisher Scientific), supplemented with a phosphatase and protease inhibitor cocktail. Protein A/G magnetic beads (MedChemExpress) were incubated with either the indicated antibody or control IgG for 2 h at 4°C. Subsequently, the samples were incubated with the protein A/G magnetic beads-Ab-Ag complex for 2 h at 4°C. After incubation, the samples were washed thrice with elution buffer and then boiled in sodium dodecyl sulfate (SDS) loading buffer.

### 2.7 Confocal microscopy and co-localization analysis

MGF360-4L-Flag and IRF3-HA expression plasmids were transfected into HEK293T cells and PK-15 cells. After 24 h of transfection, the cells were fixed with 4% paraformaldehyde for 30 min at room temperature, then permeabilized with 0.1% Triton X-100 for 15 min. Subsequently, the cells were blocked with 5% BSA for 1 h. Next, they were incubated with corresponding primary antibodies at 4°C overnight. Following this, the cells were incubated with Alexa Fluor 594 or 488-conjugated secondary antibodies for 2 h and then stained with 4-methyl-6-phenylindole for 10 min. The samples were examined using the Leica TCS SP8 confocal system (Leica Microsystems).

### 2.8 Western blot analysis

The cells were harvested and washed with cold phosphate-buffered saline, then lysed on ice with the radio immunoprecipitation assay buffer (CWBIO), supplemented with a protease and phosphatase inhibitor cocktail (CWBIO). After centrifuging the cell lysis buffer at 4°C for 10 min, we collected the lysed supernatant. Total protein content was quantified using the Pierce™ bicinchoninic acid protein assay kit (Thermo Fisher Scientific). The cell supernatants were denatured with 5× SDS-polyacrylamide gel electrophoresis loading buffer (CWBIO) for 10 min. Each sample was separated by SDS-polyacrylamide gel electrophoresis, then transferred to Immobilon-NC membranes (Millipore). The membranes were blocked with 5% (w/v) skim milk dissolved in Tris-buffered saline containing 0.1% Tween 20 at room temperature for 2 h. Incubation with the appropriate primary antibody was performed at 4°C overnight. After incubation with secondary antibodies, the membranes were visualized using the ECL western blotting substrate (Tanon).

### 2.9 Statistical analysis

All experiments were independently performed at least three times. Statistical analyses were conducted using Student’s test and one-way analysis of variance with GraphPad Prism 5.0 software. Statistical significance was defined as *, p<0.05; **, p<0.01; and ***, p<0.001.

## 3 Results

### 3.1 ASFV MGF360-4L inhibits the cGAS-STING signaling pathway

**Figure 1:**
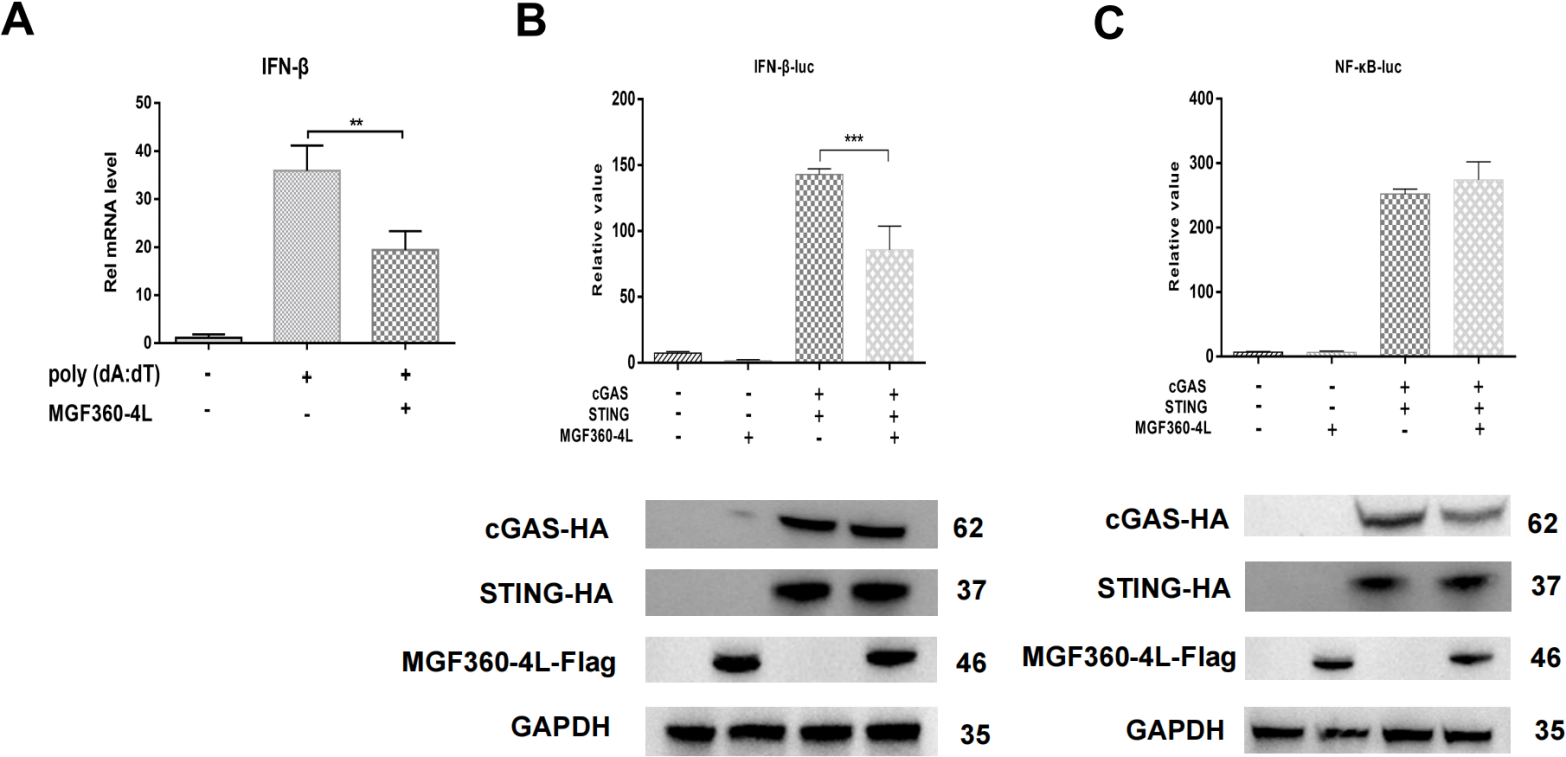
ASFV MGF360-4L suppressed IFN-β transcription mediated by the cGAS/STING pathway. **(A)** HEK293T cells were transfected with ASFV MGF360-4L-Flag, treated with 1 µg/ml poly (dA:dT), and then harvested. The IFN-β mRNA levels were then detected. **(B, C)** HEK293T cells were co-transfected with IFN-β-luc (20 ng) or NF-κB-luc (20 ng), pRL-TK (2 ng), cGAS-HA (10 ng), STING-HA (40 ng), and MGF360-4L-Flag (100 ng). After 24 h of transfection, promoter activation was detected using DLR assay kits, and the expression of cGAS-HA, STING-HA, and MGF360-4L-Flag was examined using western blot assay. **, p<0.01; and ***, p<0.001.

Previous studies in our laboratory have identified MGF360-4L as an inhibitor in the cGAS/STING-induced signaling pathways. To further confirm these results, PK-15 cells were transfected with MGF360-4L-Flag and subsequently treated with 1 µg/mL poly(dA:dT). The mRNA level of IFN-β was then detected using quantitative PCR (qPCR). As shown in **Figure 1A**, MGF360-4L significantly inhibited poly (dA:dT)-induced IFN-β mRNA expression. HEK293T cells were transfected with the MGF360-4L-Flag expression plasmid, along with either an IFN-β-luciferase or an NF-κB-luciferase reporter, pRL-TK, and cGAS-HA and STING-HA expression plasmids. At 24 h post-transfection, the cells were collected for double luciferase assay. The results showed that MGF360-4L significantly inhibited cGAS/STING-induced IFN-β promoter activation (**Figure 1B**); however, it did not inhibit NF-κB promoter activation (**Figure 1C**).

### 3.2 ASFV MGF360-4L inhibits IFN-β promoter activation and decreases IFN-β mRNA expression level in a dose-dependent manner

**Figure 2:**
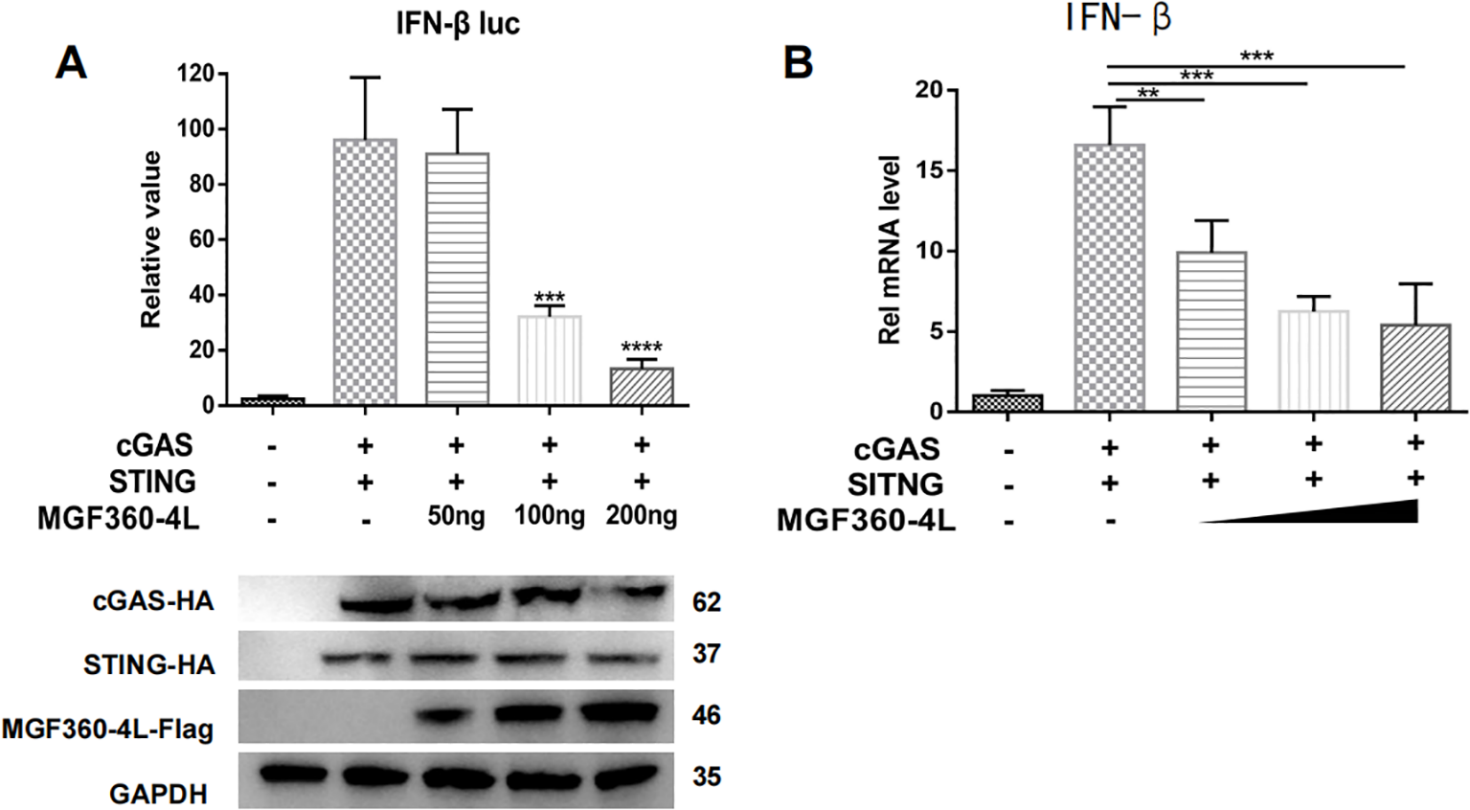
ASFV MGF360-4L inhibited IFN-β promoter activity and the mRNA levels of IFN-β in a dose-dependent manner. HEK293T cells were co-transfected with cGAS-HA, STING-HA and p3×Flag-CMV-7.1 empty vector were the control comparison group. **(A)** HEK293T cells were co-transfected with IFN-β-luc (20 ng), pRL-TK (2 ng), cGAS-HA (40 ng), and STING-HA (160 ng), along with increased amounts of ASFV MGF360-4L-flag (0 ng, 50 ng, 100 ng, 200 ng). At 24 h post-transfection, the expression of cGAS-HA, STING-HA, and MGF360-4L-Flag was detected using western blotting. Promoter activity was measured using a DLR kit. **(B)** HEK293T cells were co-transfected with cGAS-HA (40 ng) and STING-HA (160 ng), along with increased amounts of ASFV MGF360-4L-flag (0 ng, 50 ng, 100 ng, 200 ng) or the p3×Flag-CMV-7.1 empty vector, and the mRNA levels of IFN-β were then detected using qPCR. **, p<0.01; ***, p<0.001; and ****, p<0.0001.

HEK293T cells were transfected with IFN-β-luc and pRL-TK plasmids, along with cGAS-HA, STING-HA, and various doses of MGF360-4L-Flag. Through the DLR assay, the overexpression of MGF360-4L significantly inhibited cGAS/STING-induced IFN-β promoter activities in a dose-dependent manner (**Figure 2A**). The results of qPCR were consistent with those of the dual luciferase assay (**Figure 2B**).

### 3.3 ASFV MGF360-4L impairs the transcription of IFN-stimulated genes in response to 2′3′-cGAMP treatment

**Figure 3:**
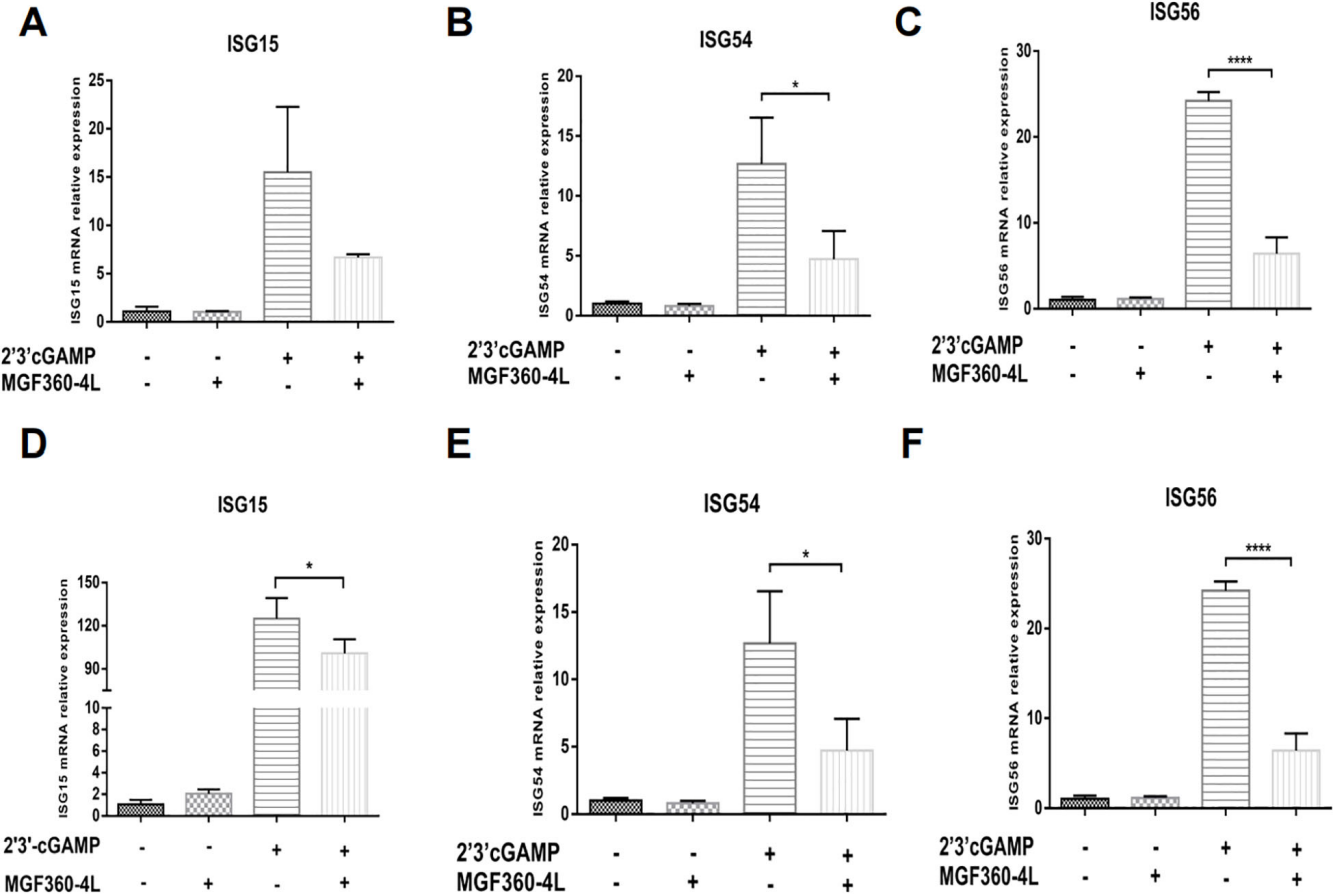
ASFV MGF360-4L reduced the mRNA levels of ISG15, ISG54 and ISG56. **(A–C)** HEK293T cells were transfected with MGF360-4L-Flag (200 ng) expression plasmid or the p3×Flag-CMV-7.1 empty vector (200 ng). At 16 h post-transfection, cells were treated with 2′3′-cGAMP (2 µg/ml) for another 12 h, then harvested and lysed, followed by RT-qPCR detection of ISG15 **(A)**, ISG54 **(B)**, and ISG56 **(C)** mRNA. **(D–F)** PK-15 cells were transfected with MGF360-4L-Flag (200 ng) expression plasmid or the p3×Flag-CMV-7.1 empty vector (200 ng). At 16 h post-transfection, cells were treated with 2′3′-cGAMP (2 µg/ml) for another 12 h, then harvested and lysed, followed by RT-qPCR detection of ISG15 **(D)**, ISG54 **(E)**, and ISG56 **(F)** mRNA. *, p<0.05; and ****, p<0.0001.

To investigate whether the MGF360-4L protein affects the expression of ISGs, we explored how IFN-β induces a series of ISGs to produce a strong antiviral response. We detected the mRNA levels of ISG15, ISG54, and ISG56 in HEK293T cells and PK-15 cells were treated with 2′3′-cGAMP (**Figures 3A–F**). The results indicated that ASFV MGF360-4L considerably inhibited the transcriptional activity of ISG54 (**Figure 3B**) and ISG56 (**Figure 3C**) in HEK 293T cells, and reduced the mRNA levels of ISG15 (**Figure 3D**), ISG54 (**Figure 3E**) and ISG56 (**Figure 3F**) in PK-15 cells. These data suggest that ASFV MGF360-4L downregulates IFN-I downstream antiviral genes.

### 3.4 ASFV MGF360-4L is involved in inhibiting the mRNA levels of STAT1, STAT2, and TYK2

**Figure 4:**
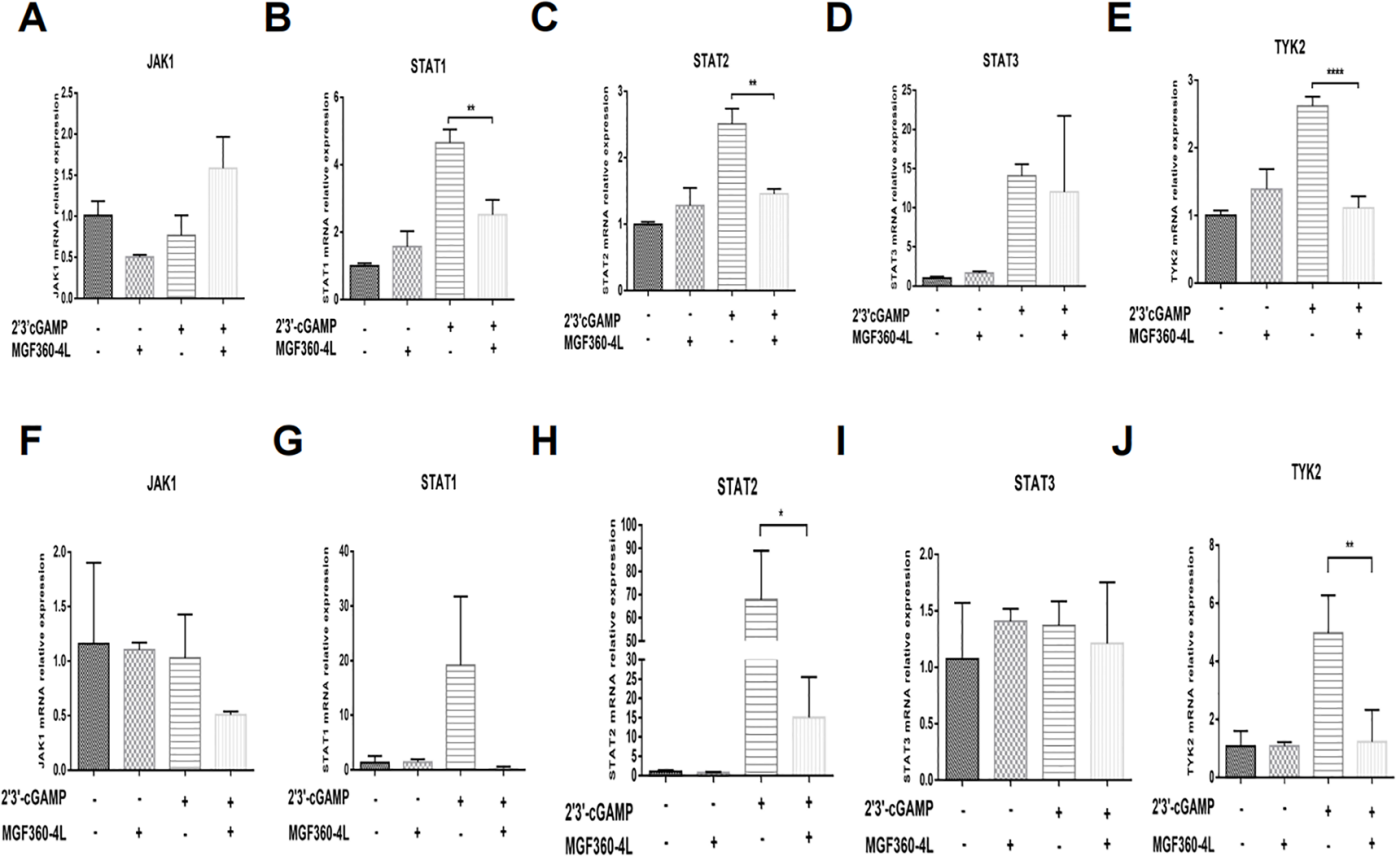
ASFV MGF360-4L suppressed the transcription levels of STAT1, STAT2, and TYK2. **(A–E)** HEK293T cells were transfected with MGF360-4L-Flag (200 ng) expression plasmid or the p3×Flag-CMV-7.1 empty vector (200 ng). At 16 h post-transfection, cells were treated with 2′3′-cGAMP (2 µg/ml) for another 12 h. Total RNA was then extracted from cell lysates, followed by RT-qPCR detection of JAK1 **(A)**, STAT1 **(B)**, STAT2 **(C)**, STAT3 **(D)**, and TYK2 **(E)** mRNA. **(F–J)** PK-15 cells were transfected with MGF360-4L-Flag (200 ng) expression plasmid or the p3×Flag-CMV-7.1 empty vector (200 ng). At 16 h post-transfection, cells were treated with 2′3′-cGAMP (2 µg/ml) for another 12 h. Total RNA was then extracted from cell lysates, followed by RT-qPCR detection of JAK1 **(F)**, STAT1 **(G)**, STAT2 **(H)**, STAT3 **(I)**, and TYK2 **(J)** mRNA. *, p<0.05; **, p<0.01; and ****, p<0.0001.

We investigated various ISGs that are induced via the Janus kinase (JAK)-signal transducer and activator of transcription (STAT) pathway (30). We found that ASFV MGF360-4L inhibited the mRNA levels of ISG15, ISG54 and ISG56. To explore the impact of MGF360-4L on JAK/STAT signaling pathways, HEK293T cells and PK-15 cells were transfected with MGF360-4L and subsequently treated with 2′3′-cGAMP. Total RNA was extracted and reverse-transcribed into cDNA. The expression of JAK1, TYK2, STAT1, STAT2, and STAT3 was evaluated by qPCR (**Figures 4A–J**). The results indicated that ASFV MGF360-4L significantly reduced the transcription levels of STAT1 (**Figure 4B**), STAT2 (**Figure 4C**), and TYK2 (**Figure 4E**) in HEK 293T cells, and inhibited the transcriptional activity of STAT2 (**Figure 4H**) and TYK2 (**Figure 4J**) in PK-15 cells. We speculated that ASFV MGF360-4L may negatively regulate the JAK/STAT signaling pathway.

### 3.5 ASFV MGF360-4L can disturb the cGAS/STING signaling mediated antiviral responses

**Figure 5:**
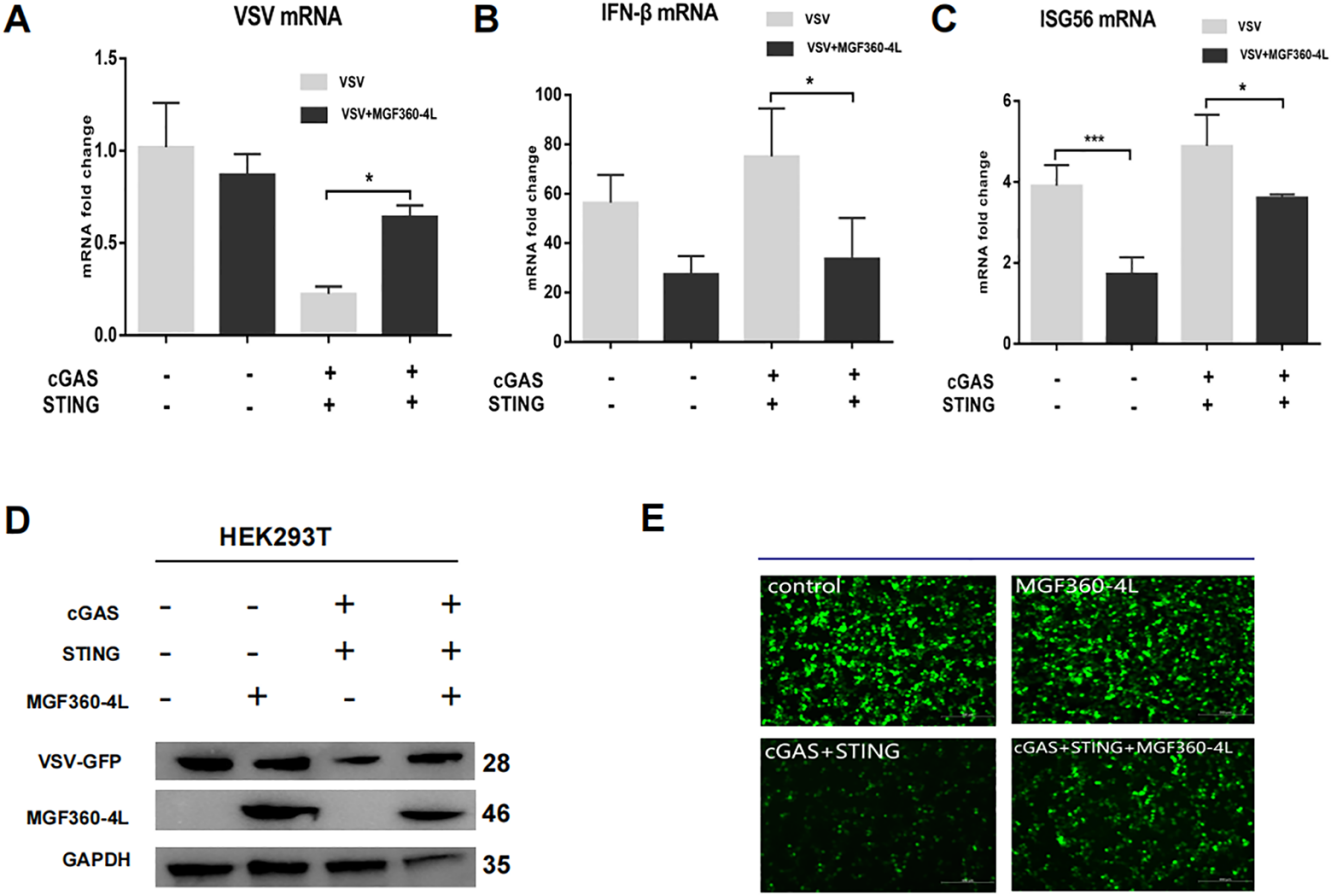
Effects of MGF360-4L on the cGAS/STING signaling mediated anti-VSV activity. **(A–E)** HEK293T cells were cultured overnight to 80% confluence. They were then co-transfected with cGAS-HA (40 ng), STING-HA (160 ng), and MGF360-4L-Flag (200 ng) for 16 h. The cells were infected with VSV-GFP for 8 h. **(A–C)** The mRNA levels of VSV, INF-β, and ISG56 were detected using RT-qPCR. The GFP signals from the VSV replicates were observed using fluorescence microscopy **(E)** and analyzed using western blotting **(D)**. *, p<0.05; and ***, p<0.001.

The cGAS/STING signaling pathway has broad antiviral functions (31); thus, we wanted to determine whether ASFV MGF360-4L inhibits cGAS/STING mediated antiviral responses. Therefore, the HEK293T cells were infected with VSV-GFP, which stably expresses green fluorescent protein. In the presence of ASFV MGF360-4L, the transcription and expression levels of VSV-GFP were significantly increased (**Figures 5A, D**), which was evidenced by fluorescence microscopy (**Figure 5E**). In the presence of cGAS-HA, STING-HA, and MGF360-4L-Flag, the mRNA levels of IFN-β and ISG56 were significantly reduced (**Figures 5B, C**), suggesting that ASFV MGF360-4L may inhibit the cGAS/STING signaling pathway. Taken together, these data indicate that ASFV MGF360-4L potentially inhibits the cGAS/STING signaling mediated anti-VSV responses.

### 3.6 ASFV MGF360-4L inhibits the cGAS/STING pathway by suppressing IRF3 phosphorylation

**Figure 6:**
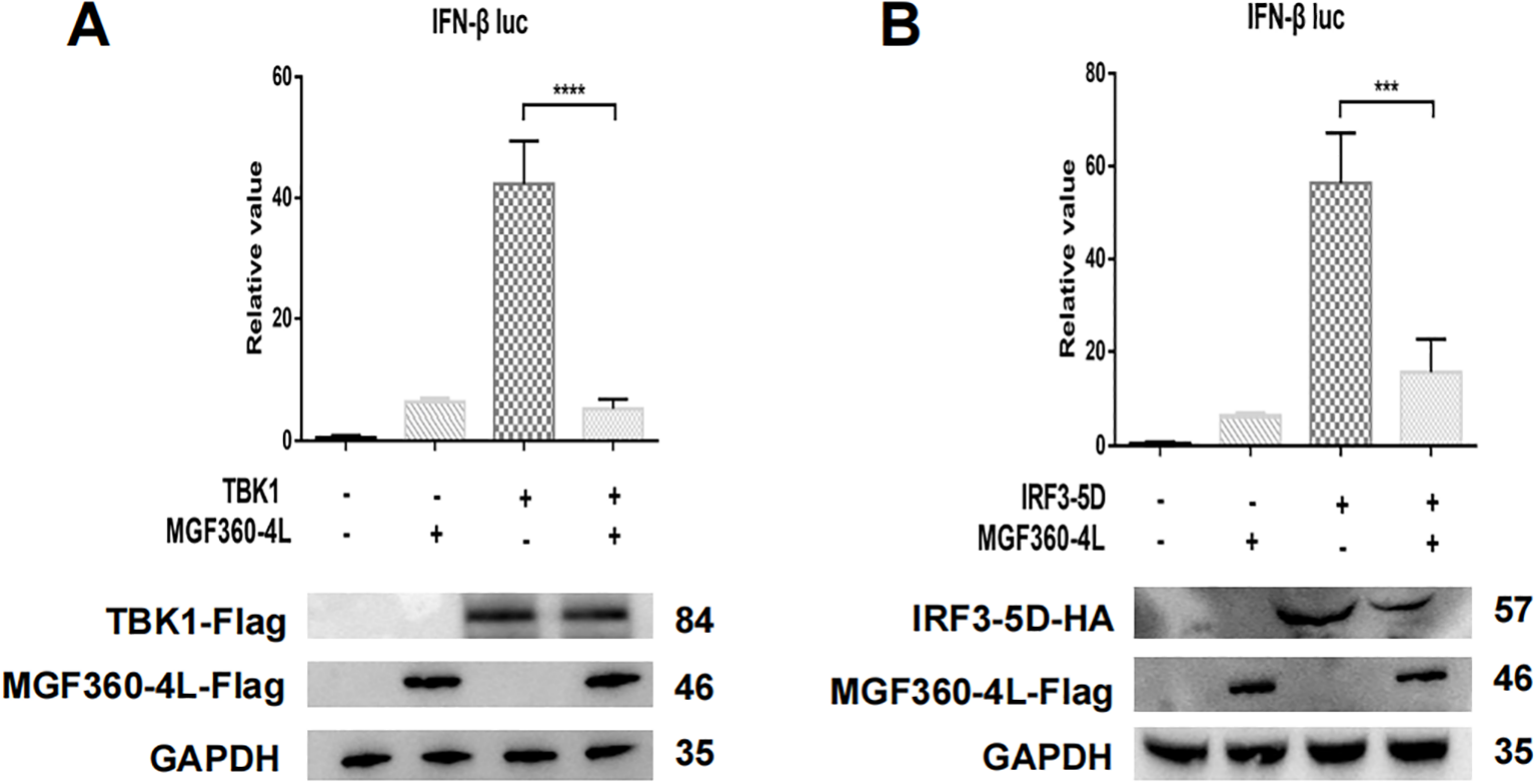
ASFV MGF360-4L inhibited the IFN- β promoter activity induced by TBK1 and IRF3-5D. **(A, B)** HEK 293T cells were co-transfected with IFN-β-luc (20 ng), pRL-TK (2 ng), MGF360-4L-Flag (200 ng) and TBK1-Flag (200 ng) or IRF3-5D-HA (200 ng), then harvested and lysed. Promoter activation was detected using DLR assay kits, and the expression of cGAS-HA, STING-HA, and MGF360-4L was examined using western blot assay. ***, p<0.001, and ****, p<0.0001.

**Figure 7:**
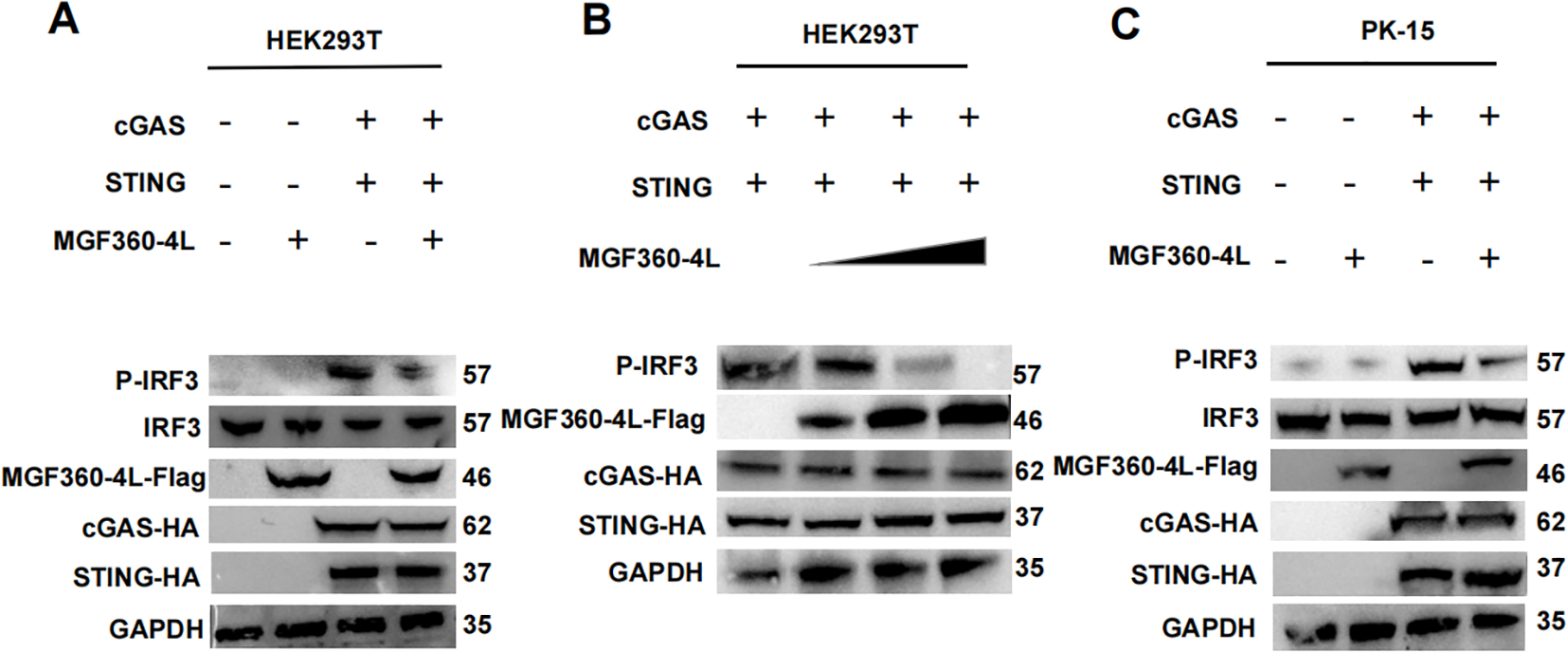
ASFV MGF360-4L inhibited the cGAS/STING pathway by suppressing the phosphorylation of IRF3. **(A)** HEK293T cells were co-transfected with cGAS-HA (40 ng), STING-HA (160 ng) and ASFV MGF360-4L-Flag (200 ng). After 24 h of transfection, the expression of cGAS-HA, STING-HA, and MGF360-4L-Flag was examined using western blot assay. **(B)** HEK293T cells were co-transfected with cGAS-HA (40 ng), and STING-HA (160 ng), along with increased amounts of ASFV MGF360-4L-flag (0 ng, 50 ng, 100 ng, 200 ng). At 24 h post-transfection, the expression of cGAS-HA, STING-HA, and MGF360-4L-Flag was detected using western blotting. **(C)** PK-15 cells were co-transfected with cGAS-HA (40 ng), STING-HA (160 ng) and ASFV MGF360-4L-Flag (200 ng). After 24 h of transfection, the expression of cGAS-HA, STING-HA, and MGF360-4L-Flag was examined using western blot assay.

To explore the molecular mechanism underlying the ASFV MGF360-4L inhibition of the cGAS/STING pathway, HEK293T cells were transfected. The transfection involved a plasmid TBK1-Flag or an HA-tagged, active mutant of IRF3 (IRF3-5D-HA), along with an IFN-β-luc, pRL-TK, and MGF360-4L-Flag. The results showed that ASFV MGF360-4L inhibited the IFN-β promoter activity, which was induced by TBK1 (**Figure 6A**) and IRF3-5D (**Figure 6B**). Furthermore, it inhibited the expression of the IRF3-5D protein (**Figure 6B**), while the exogenous expression of TBK1 remained unchanged (**Figure 6A**). Further research showed that ASFV MGF360-4L inhibited the phosphorylation of IRF3 induced by cGAS/STING (**Figure 7A**), without altering the endogenous protein levels of IRF3, we transfected different dose of ASFV MGF360-4L, the results showed that the overexpression of MGF360-4L suppressed the phosphorylation of IRF3 induced by cGAS/STING in a dose-dependent manner (**Figure 7B**). At the same time, we also proved that MGF360-4L could suppress the phosphorylation of IRF3 in PK-15 cells (**Figure 7C**). These results suggest that ASFV MGF360-4L inhibits the cGAS/STING signaling pathway by suppressing IRF3 phosphorylation.

### 3.7 ASFV MGF360-4L interacts with IRF3

**Figure 8:**
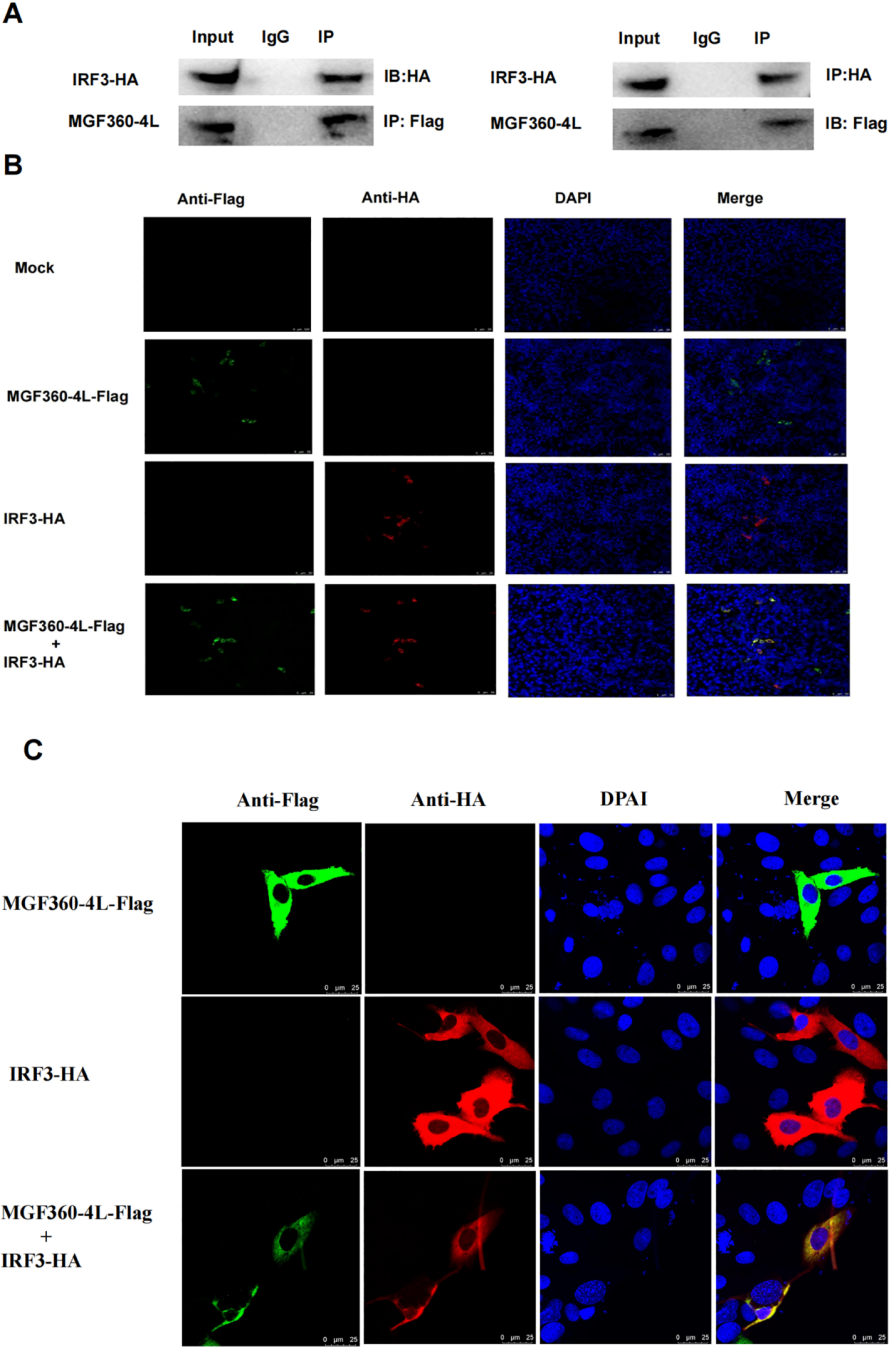
MGF360-4L interacted and co-localized with IRF3. **(A)** MGF360-4L interacted with IRF3 in the overexpression system. HEK 293T cells were transfected with the indicated plasmids (5 µg each). IgG was used for control immunoprecipitation. Co-immunoprecipitation and immunoblotting analyses were performed with the indicated Abs. **(B, C)** Co-localization of MGF360-4L with IRF3. HEK 293T **(B)** cells and PK-15 cells **(C)** were transfected with MGF360-4L-Flag and IRF3-HA expression plasmids. After 24 h of transfection, MGF360-4L-Flag and IRF3-HA were stained with mouse-anti-Flag (green) and rabbit-anti-HA (red), and the nuclei were stained with DAPI (blue). Confocal assays were performed using Leica TCS SP8.

Previous studies have proven that several viral proteins antagonize IFN-I production by interacting with IRF3 (32). To determine whether MGF360-4L interacts with IRF3, HEK293T cells were co-transfected with MGF360-4L-Flag and IRF3-HA expression plasmids. Co-immunoprecipitation results confirmed the interaction between MGF360-4L and IRF3 (**Figure 8A**). We also found that MGF360-4L co-localized with IRF3 in the cytoplasm when the two proteins were co-expressed in HEK293T cells and PK-15 cells (**Figures 8B, C**), These results show that MGF360-4L could interact and co-localize with IRF3.

### 3.8 ASFV MGF360-4L regions are responsible for inhibitory activity

**Figure 9:**
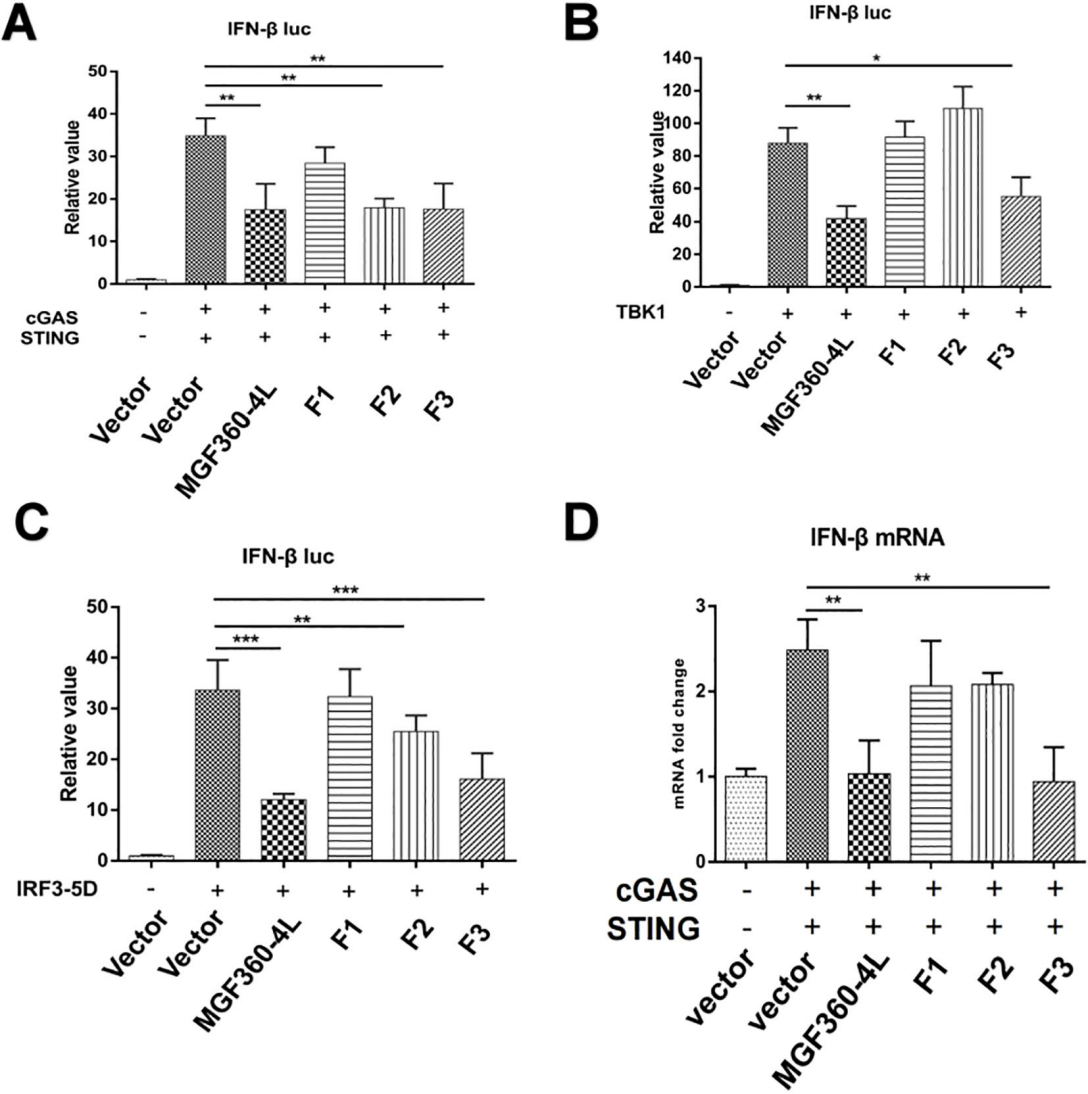
Regions of ASFV MGF360-4L responsible for inhibitory activity. **(A–C)** Luciferase assays in HEK293T cells were performed to detect the activation of the IFN-β promoter following the expression of empty vector, cGAS-HA and STING-HA, TBK1-Flag, or IRF3-5D-HA in the presence of empty vector, and full-length MGF360-4L-Flag, 4L-F1-Flag (1-88 aa), 4L-F2-Flag (75-162 aa), or 4L-F3-Flag (146-387 aa) plasmids. **(D)** HEK293T cells were co-transfected with cGAS-HA, STING-HA, and full-length MGF360-4L-Flag, 4L-F1-Flag (1-88 aa), 4L-F2-Flag (75-162 aa), or 4L-F3-Flag (146-387 aa) plasmids. At 24 h post-transfection, the mRNA levels of IFN-β were detected using RT-qPCR. *, p<0.05; **, p<0.01; and ***, p<0.001.

To identify the crucial domains in ASFV MGF360-4L that are related to its immunosuppressive function, three truncated mutants of MGF360-4L, namely 4L-F1 (1-88 aa), 4L-F2 (75-162 aa), and 4L-F3 (146-387 aa), were constructed. We found that 4L-F2 inhibited the IFN-β promoter activity induced by cGAS/STING and IRF3-5D (**Figures 9A, C**), 4L-F3 significantly inhibited the IFN-β promoter activity induced by cGAS/STING, TBK1, and IRF3-5D (**Figures 9A–C**), and 4L-F3 reduced the mRNA level of IFN-β (**Figure 9D**). Thus, we speculate that the domains and sites within 4L-F2 and 4L-F3 are responsible for immunosuppressive effects.

## 4 Discussion

IFN-I represents the most effective mechanism through which the host exerts innate immunity against viral infections (33). Upon infection with DNA viruses, the viral DNA can be sensed by the host cytosolic DNA sensor cGAS, initiating a series of antiviral signaling pathways that result in the induction of IFN-β and ISG expression to combat viral infections. Many viruses have taken multiple strategies to evade host immune defenses by inhibiting the cGAS/STING signal pathway. Foot-and-Mouth Disease Virus (FMDV) and Senecavalley virus (SVV) attenuate IFN-I production by inhibiting the expression of the IRF3 protein (34, 35). Human cytomegalovirus facilitates the proteasome-dependent degradation of STING and blocks the cGAS/STING pathway (36).

**Figure 10:**
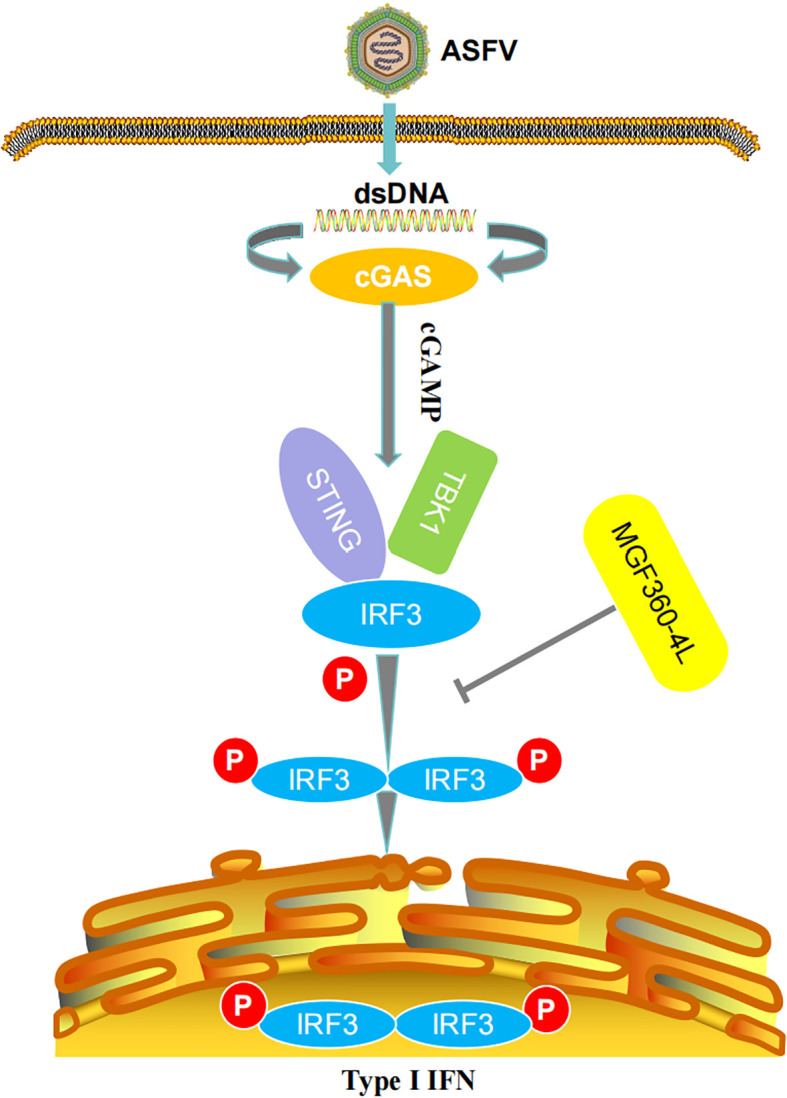
Schematic diagram of the mechanism of MGF360-4L-mediated IFN-β signaling inhibition. ASFV MGF360-4L interacted with IRF3 and suppressed the phosphorylation of IRF3 to help the virus escape the host immune response.

Increasing evidence has shown that ASFV has developed a series of strategies to reduce IFN production and inhibit IFN-I signaling pathways. For instance, ASFV pE120R interferes with the interaction between IRF3 and TBK1, thereby inhibiting the activation of IRF3 and suppressing the production of IFN-I (24). ASFV pI215L inhibits the production of IFN-I by recruiting the E3 ubiquitin ligase RNF138 to facilitate the degradation of RNF128, thereby inhibiting RNF128-mediated k63-linked polyubiquitination of TBK1 (37). Preventing the antiviral immune response is crucial for the proliferation of ASFV. Probably for this reason, the ASFV genome encodes several proteins dedicated to the control of IFN-I, mainly within the MGF360 and MGF505/530 multigene families, aiming to evade host defenses (29, 38–40). For example, ASFV pMGF505-7R reduces IFN-I production by promoting ULK1-mediated STING degradation and inhibiting IRF3 phosphorylation and nuclear translocation (41). ASFV MGF360-14L negatively regulates IFN-I signaling by targeting IRF3 (22). ASFV MGF360-4L is a member of MGF360 multigene families, while whether ASFV MGF360-4L could inhibit IFN-I signaling pathway and the underlying molecular mechanisms IFN-I remain unknown. In our study, ASFV MGF360-4L was firstly identified as a new inhibitor of IFN-I induced by cGAS/STING pathway (**Figure 10**). Firstly, ASFV MGF360-4L inhibited IFN-β promoter activity induced by cGAS/STING, TBK1, and IRF3-5D, reduced the mRNA levels of IFN-β, ISG15, ISG54, and ISG56, and might antagonize the JAK/STAT signaling pathway. Second, ASFV MGF360-4L disrupted the cGAS/STING signaling mediated antiviral responses, leading to increased yields of VSV-GFP in cells transfected with cGAS, STING, and MGF360-4L compared with cells transfected with cGAS and STING. Third, ASFV MGF360-4L could interact with IRF3, and suppressed the phosphorylation of IRF3, thereby blocking the cGAS/STING signaling pathway. IRF3 is a key transcription factor capable of inducing IFN-I and facilitating the expression of numerous genes involved in the innate immune response (42). Fourth, we identified crucial domains related to immunosuppressive function through DLR and qPCR. We speculated that 4L-F2 (75-162 aa) and 4L-F3 (146-387 aa) contain immunosuppressive domains and sites.

ASFV has caused severe economic losses to the global pig industry; thus, investigating the functions of ASFV proteins and elucidating the immune evasion mechanism are needed. In this study, we found that ASFV MGF360-4L suppressed the phosphorylation of IRF3, thereby inhibiting the type I IFN response induced by cGAS/STING. This study provides a novel strategy for understanding ASFV-mediated immune evasion mechanisms.

## Data Availability

The original contributions presented in the study are included in the article/supplementary material. Further inquiries can be directed to the corresponding authors.
